# Osteopathic Manipulative Treatment During Post-operative Recovery: A Scoping Review

**DOI:** 10.7759/cureus.54233

**Published:** 2024-02-15

**Authors:** Chandler G Randall, Heather A Paul, Heather Lumley, Angelica Ortega, Jace Rowley, Bailey Brown, Sukanya Mohan, Kristina Smith, Thomas Messer, Emily Swan, Rohit S Mehra

**Affiliations:** 1 Osteopathic Medicine, Nova Southeastern University Dr. Kiran C. Patel College of Osteopathic Medicine, Clearwater, USA; 2 Osteopathic Medicine, Nova Southeastern University Dr. Kiran C. Patel College of Osteopathic Medicine, Fort Lauderdale, USA

**Keywords:** hands-on approach, osteopathic manipulative treatment, osteopathic manipulative medicine, post-surgical, post-operative

## Abstract

Surgery is a common and often necessary treatment option for a wide range of medical conditions, with an estimated 40 to 50 million surgeries performed in the US alone each year. While the various types of surgeries performed may be effective in treating or managing different conditions, the post-operative period can be challenging for patients. Osteopathic manipulative treatment (OMT) is a hands-on approach to medical care that seeks to restore balance and harmony to the body from the lens of an interconnected mind, body, and spirit. Given the potential for adverse events in patients following surgical treatments, OMT may be a viable adjunct post-operatively to enhance patient care and recovery. The purpose of this scoping review is to evaluate the state of current research examining the effectiveness of OMT in improving outcomes in post-operative patients. Three hundred articles were collected; 53 duplicates were removed. Eleven independent reviewers evaluated all 247 articles. Thirty articles were identified, including nine in general surgery, six in cardiothoracic surgery, five in orthopedic surgery, four in spinal surgery, three in neurosurgery, and three others (otolaryngology, oral/maxillofacial, and gynecologic surgery). Post-operative patients were treated with various OMT techniques with myofascial release and muscle energy being some of the most common treatments utilized in all surgical fields. Many studies demonstrated the benefits of OMT usage including significant pain relief, improved and earlier bowel function, and decreased lengths of hospital stay. This study demonstrates how OMT can be effective in reducing post-operative pain, reducing the incidence of post-operative ileus, and shortening the length of stay. Further research into the utilization of OMT in post-operative patients should be considered a potential adjunct to surgical intervention, especially in vulnerable patient populations.

## Introduction and background

Osteopathic manipulative medicine (OMM) involves a subset of medicine that aims to correct somatic dysfunctions from the focus of an interconnected body, mind, and spirit [1,2]. Somatic dysfunction refers to impaired or altered function in the body’s framework and is associated with TART changes: Tissue texture change, Asymmetry, Range of motion decrease, and Tenderness [3,4]. Often, patients present with overlapping somatic dysfunctions, leading to pain and symptoms in more than one part of the body [1-3]. Somatic dysfunctions can arise during the post-operative period due to the surgical manipulation of tissues [5]. Surgical procedures can also cause physiologic derangements and anatomic or postural changes as compensatory mechanisms to address the new state the body is in [5]. Some of the causes of post-operative pain include a variety of factors: incisional pain, nerve injury or entrapment, pneumoperitoneum, intraoperative positioning, tissue manipulation, and metabolic changes, among others [6].

It is estimated that approximately 312 million surgical operations are performed worldwide each year [7]. With regards to post-operative recovery, osteopathic manipulative therapy (OMT) is the therapy utilized to address found somatic dysfunctions [6]. It may be utilized to help promote the self-healing of a patient to reduce pain, minimize the usage of analgesics, increase limited range of motion, increase blood flow and lymphatic circulation, and overall return the patient to homeostasis [1-3,8]. Overall, OMT can potentially be utilized to allow patients greater ability to complete activities of daily living (ADLs) [5].

Osteopathic physicians evaluate and treat a variety of different body systems with OMT corresponding to various viscerosomatic reflexes that may be compromised during these surgical procedures [9]. Treatment of these specific viscerosomatic points in the post-operative period can be performed either as a solo technique or a combination of techniques that include high-velocity low amplitude (HVLA), facilitated positional release (FPR), muscle energy (ME), and myofascial release (MFR) techniques [3].

Several studies have been conducted to examine the potential outcomes of utilizing OMT as a part of therapy for various surgical procedures. A variety of different body systems and surgical techniques have been researched including musculoskeletal, cardiac, pulmonary, oncologic, and more [1,5,10-36]. These studies have demonstrated decreased symptoms at viscerosomatic levels of treatment [19,25], improved lymphatic circulation [37], improved cardiac function and perfusion [26], decreased length of hospital stay [11,14,29,35], decreased pain [20,21,24,28,29,31,32,34,36,38], decreased pain medication use [1,22,28,39] and decreased morbidity [29].

Many studies focus on the use of OMT in reference to pain relief, but only a few focus on the use of OMT in aiding surgical patient recovery [8,40]. Studies have demonstrated that although OMT is beneficial in improving musculoskeletal pain symptoms, the frequency of OMT use is low, which may suggest many physicians are unaware or uninformed of the potential benefits [41]. In addition, a study utilizing patient surveys revealed an overwhelming majority of patients felt that OMT was helpful for their own recovery and would recommend OMT as part of other patients' recovery and treatment plans during hospitalizations [28].

This review aims to explore the current literature on the usage and results of OMT in all post-operative patient management, as well as identify how the use of OMT could be implemented into a more standard practice of post-operative care.

## Review

Methods 

Identifying the Research Question

The research question was based on the Population, Concept, and Context (PCC) strategy: population included patients of any age, concept included post-operative patients in the setting of a hospital, clinic, or rehabilitation facility, and context included the usage of OMT in post-operative patients. Through this strategy, the review question was “What are the benefits of OMT in post-operative patients?” 

Identifying Relevant Studies

A search was conducted of EMBASE, CINAHL, Biomedical Reference Collection: Comprehensive, Nursing & Allied Health Collection: Comprehensive, and MEDLINE with full text to include citations from inception to September 19, 2022. Authors CR, HP, and HL, each did an initial search independently utilizing the same controlled terms, as outlined in Table 1, to broaden the search and ensure consistency.

**Table 1:** Boolean operators utilized in search

Search Queries	
Number (No.)	Query
#6	postoperati*:ab,ti,kw OR 'post surg*':ab,ti,kw OR postsurg*:ab,ti,kw
#5	postoperative period'/exp
#4	('manipulative medicine':ab,ti,kw OR 'soft tissue therapy':ab,ti,kw OR 'musculoskeletal manipulation':ab,ti,kw OR 'trigger point therapy':ab,ti,kw OR omm:ab,ti,kw OR omt:ab,ti,kw) AND osteopath*:ab,ti,kw
#3	trigger point therapy'/exp
#2	soft tissue therapy'/exp
#1	osteopathic medicine'/exp

Selecting Studies

Our inclusion criteria included articles that were peer-reviewed, written, and published in English, and included the usage of osteopathic manipulative treatment (OMT) techniques in post-operative patients. Research studies from any time period were included in this review. Articles were excluded if the techniques involved in patient treatment were acupuncture, physical therapy, chiropractic, other non-specified osteopathic manipulation techniques, or not performed by an osteopath. For the purpose of this study, an osteopath is an osteopathic physician or osteopathic medical student overseen by an osteopathic physician. Studies where patients did not receive treatment in the post-operative period were also excluded. These exclusions were created to ensure the focus was on the improvement of post-operative patients through the usage of OMT. To achieve a scoping review, editorial papers, systematic reviews, meta-analyses, and other scoping review studies were excluded. To further ensure consistency in our review, articles that were translated into English were excluded.

Charting the Data

A data charting format was developed by authors CR, HP, and HL to determine which variables to extract from each article reviewed. The variables included the number of participants, demographics, OMT techniques utilized, time range, OMT usage in post-operative long-term complications, outcomes, and limitations. The team of 11 researchers was then divided into groups of two to analyze the remaining 30 articles. A third reviewer was implemented to resolve discrepancies. Then, CR, HP, and HL independently charted the data, discussed the results to resolve inconsistencies, and continuously updated the data-charting form in a uniform process.

Data were abstracted on article characteristics (i.e., type of study, the time frame of publication), surgical field (i.e., general, cardiothoracic, orthopedic), type of surgery performed, timeline post-surgery, OMT techniques used (i.e., HVLA, ME, MFR, CS, BLT), and results of OMT usage, including both physician assessment and patient reported outcomes.

Collating, Summarizing, and Reporting the Results

Quality analysis was utilized following the tier 2 review to assess the bias and content of each selected article. Authors CR, HP, and HL reviewed each article utilizing the Joanna Briggs Institute (JBI) Appraisal Tools. With the utilization of this resource, alongside the inclusion/exclusion criteria articles were further determined to be included or excluded. The JBI assessed the risk of bias and was able to classify articles with a high risk of bias (those scoring less than 50%), moderate bias (50%-70%), and low risk of bias (scores above 70%). Studies scoring moderate or low risk of bias were included in this study, and any scoring high risk was excluded.

Studies were grouped by time frame and surgical field to better synthesize the range of evidence answering our research questions and objectives. The information was summarized in a narrative format and described the settings, populations, and study designs, along with the measures used and broad findings.

Results 

Selection of Sources of Evidence 

The initial search identified 300 citations. First, 12 duplicates were removed via automation, and another 41 duplicates were removed by reviewers. All 11 authors evaluated every title and abstract of 247 articles through the protocol criteria. Then authors CR, HP, and HL discussed the disputed articles for final inclusion in the review. From the 247 articles, 209 articles were excluded for not following the screening criteria: 33 studies were the wrong type of study, 13 studies were not published in English, 91 studies did not include OMT techniques, and 67 studies did not evaluate post-operative patients. 

**Figure 1:**
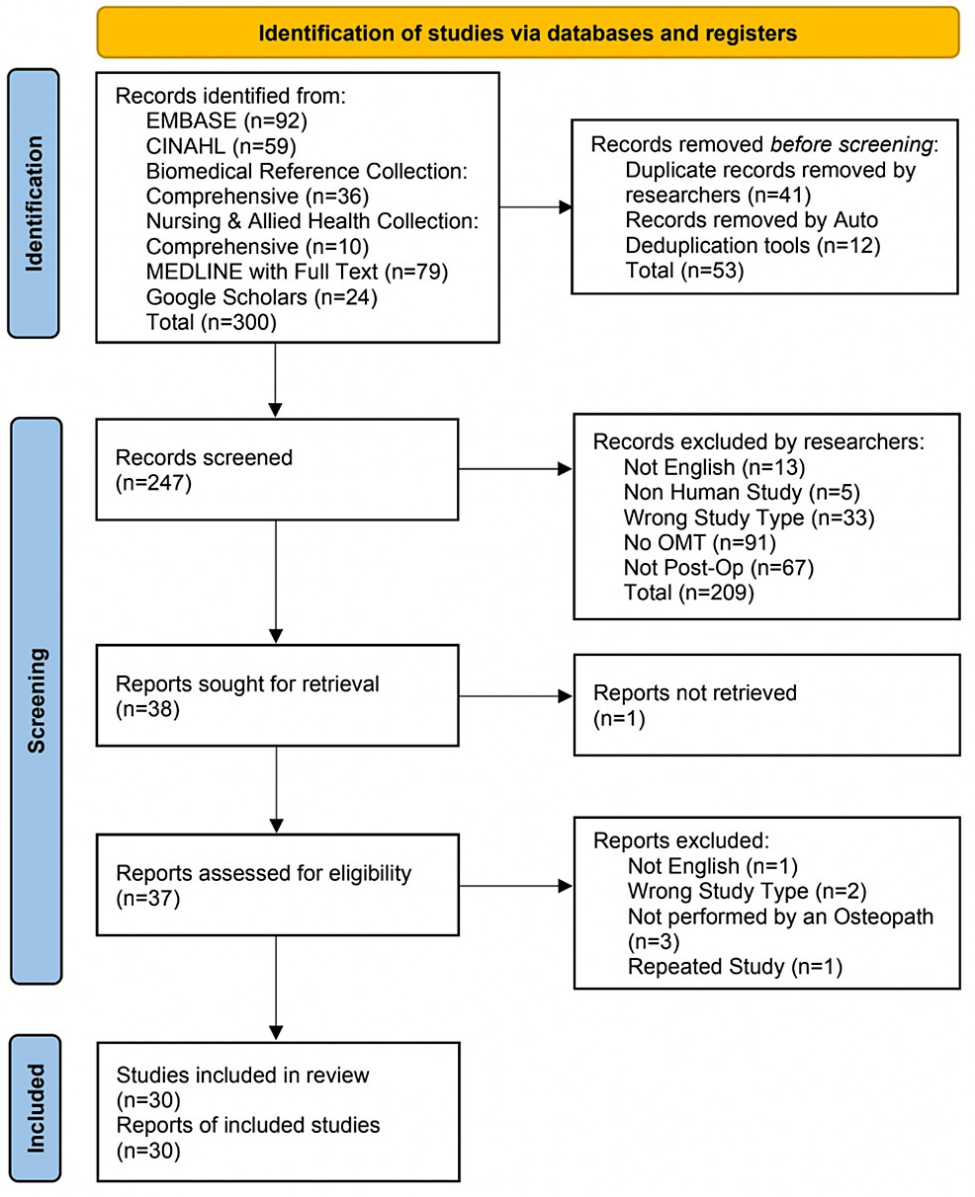
PRISMA flow diagram PRISMA: Preferred Reporting Items for Systematic Reviews and Meta-Analyses

In conclusion, 38 articles were retrieved for analysis. The full text for one article was unable to be obtained prior to the tier 2 review. There were 37 articles assessed for eligibility during the tier 2 review and seven articles were excluded. One study was not published in English, two studies were the wrong type of study, three studies were not performed by an osteopath, and one study was a repeated study. Our protocol was drafted using the Preferred Reporting Items for Systematic Reviews and Meta-analysis Protocols extension for Scoping Reviews (PRISMA-ScR) 2020 form. The screening process and flow are shown in the PRISMA flowchart in Figure 1.

Critical Appraisal Within Sources of Evidence

Each article was analyzed through the type of study and the bias relevant to that study type as seen in Tables 2-7. Articles within the moderate risk or low risk of bias were accepted, given they followed the inclusion criteria.

**Table 2:** JBI critical appraisal checklist for case reports JBI - Joanna Briggs Institute

Article	Were patient’s demographic characteristics clearly described?	Was the patient’s history clearly described and presented as a timeline?	Was the current clinical condition of the patient on presentation clearly described?	Were diagnostic tests or assessment methods and the results clearly described?	Was the intervention(s) or treatment procedure(s) clearly described?	Was the post-intervention clinical condition clearly described?	Were adverse events (harms) or unanticipated events identified and described?	Does the case report provide takeaway lessons?	% Appraisal
Arnold et al., 2010 [10]	Yes	Yes	Yes	Yes	Yes	No	No	Yes	75%
Berkowitz, 2014 [12]	Yes	Yes	Yes	Yes	Yes	Yes	Yes	Yes	100%
Dhanasekar et al., 2006 [15]	Yes	Yes	Yes	Yes	Yes	Yes	Yes	Yes	100%
Domalski et al., 2014 [16]	Yes	No	Yes	Yes	Yes	No	No	Yes	63%
Gugel et al., 2006 [19]	Yes	Yes	Yes	Yes	Yes	Yes	Yes	Yes	100%
Ivanov et al., 2016 [20]	Yes	Yes	Yes	Yes	Yes	Yes	No	Yes	88%
Lewis, 2018 [23]	Yes	Yes	Yes	Yes	Yes	No	No	Yes	75%
Lipton et al., 2013 [24]	Yes	Yes	Yes	Yes	Yes	Yes	Yes	Yes	100%
Noblitt et al., 2019 [25]	Yes	Yes	Yes	Yes	Yes	Yes	Yes	Yes	100%
Petree et al., 2015 [27]	Yes	Yes	Yes	Yes	Yes	Yes	No	Yes	88%
Ridgeway et al., 2010 [31]	Yes	Yes	Yes	Yes	Yes	Yes	Yes	Yes	100%
Shiu et al., 2012 [33]	Yes	Yes	Yes	No	Yes	No	No	Yes	63%
Vismara et al., 2020 [34]	Yes	Yes	Yes	Yes	Yes	Yes	No	Yes	88%
Zegarra-Parodi et al., 2010 [36]	Yes	Yes	Yes	Yes	No	No	No	Yes	63%

**Table 3:** JBI critical appraisal checklist for randomized controlled trials JBI - Joanna Briggs Institute

Article	Was true randomization used for assignment of participants to treatment groups?	Was allocation to treatment groups concealed?	Were treatment groups similar at the baseline?	Were participants blind to treatment assignment?	Were those delivering treatment blind to treatment assignment?	Were outcomes assessors blind to treatment assignment?	Were treatment groups treated identically other than the intervention of interest?	Was follow up complete and if not, were differences between groups in terms of their follow up adequately described and analyzed?	Were participants analyzed in the groups to which they were randomized?	Were outcomes measured in the same way for treatment groups?	Were outcomes measured in a reliable way?	Was appropriate statistical analysis used?	Was the trial design appropriate, and any deviations from the standard RCT design (individual randomization, parallel groups) accounted for in the conduct and analysis of the trial?	% Appraisal
Licciardone et al., 2004 [1]	Yes	Yes	Yes	Yes	Yes	Yes	Yes	Yes	Yes	Yes	Yes	Yes	Yes	100%
Goldstein et al., 2005 [18]	Yes	Yes	Yes	Yes	Yes	Yes	Yes	Yes	Yes	Yes	Yes	Yes	Yes	100%
Kim et al., 2017 [21]	Yes	Yes	Yes	No	No	No	Yes	Yes	Yes	Yes	Yes	Yes	Yes	77%
Kim et al., 2019 [22]	Yes	No	Yes	No	No	No	Yes	Yes	Yes	Yes	Yes	Yes	Yes	69%
Probst et al., 2016 [29]	Yes	Yes	Yes	No	No	No	Yes	Yes	Yes	Yes	Yes	Yes	Yes	77%
Roncada, 2020 [32]	Yes	Yes	Yes	No	No	Yes	Yes	Yes	Yes	Yes	Yes	Yes	Yes	85%
Wieting et al., 2013 [35]	Yes	Yes	Yes	Yes	Yes	Yes	Yes	Yes	Yes	Yes	Yes	Yes	Yes	100%
Kim et al., 2015 [38]	Yes	Yes	Yes	No	No	Yes	Yes	Yes	Yes	Yes	Yes	Yes	Yes	85%
Kim et al., 2016 [39]	Yes	Yes	Yes	No	No	No	Yes	Yes	Yes	Yes	Yes	Yes	Yes	77%

**Table 4:** JBI critical appraisal checklist for case series JBI - Joanna Briggs Institute

Article	Were there clear criteria for inclusion in the case series?	Was the condition measured in a standard, reliable way for all participants included in the case series?	Were valid methods used for identification of the condition for all participants included in the case series?	Did the case series have consecutive inclusion of participants?	Did the case series have complete inclusion of participants?	Was there clear reporting of the demographics of the participants in the study?	Was there clear reporting of clinical information of the participants?	Were the outcomes or follow up results of cases clearly reported?	Was there clear reporting of the presenting site(s)/clinic(s) demographic information?	Was statistical analysis appropriate?	% Appraisal
Mills et al., 2020 [5]	Yes	Yes	Yes	Yes	Yes	Yes	Yes	Yes	Yes	Yes	100%
Crow et al., 2009 [14]	Yes	Yes	Yes	Yes	Yes	Yes	Yes	Yes	Yes	Yes	100%
Fleming et al., 2015 [17]	Yes	Yes	Yes	Yes	Yes	Yes	Yes	Yes	Yes	Yes	100%

**Table 5:** JBI critical appraisal checklist for quasi-experimental studies (non-randomized experimental studies) JBI - Joanna Briggs Institute

Article	Is it clear in the study what is the ‘cause’ and what is the ‘effect’ (i.e. there is no confusion about which variable comes first)?	Were the participants included in any comparisons similar?	Were the participants included in any comparisons receiving similar treatment/care, other than the exposure or intervention of interest?	Was there a control group?	Were there multiple measurements of the outcome both pre and post the intervention/exposure?	Was follow up complete and if not, were differences between groups in terms of their follow up adequately described and analyzed?	Were the outcomes of participants included in any comparisons measured in the same way?	Were outcomes measured in a reliable way?	Was appropriate statistical analysis used?	% Appraisal
Bjersa et al., 2013 [13]	Yes	Yes	Yes	No	Yes	No	Yes	Yes	Yes	78%
O-Yurvati et al., 2005 [26]	Yes	Yes	Yes	Yes	Yes	No	Yes	Yes	Yes	89%

**Table 6:** JBI critical appraisal checklist for cohort studies JBI - Joanna Briggs Institute

Article	Were the two groups similar and recruited from the same population?	Were the exposures measured similarly to assign people to both exposed and unexposed groups?	Was the exposure measured in a valid and reliable way?	Were confounding factors identified?	Were strategies to deal with confounding factors stated?	Were the groups/participants free of the outcome at the start of the study (or at the moment of exposure)?	Were the outcomes measured in a valid and reliable way?	Was the follow up time reported and sufficient to be long enough for outcomes to occur?	Was follow up complete, and if not, were the reasons to loss to follow up described and explored?	Were strategies to address incomplete follow up utilized?	Was appropriate statistical analysis used?	% Appraisal
Baltazar et al., 2013 [11]	Yes	Yes	Yes	No	No	Yes	Yes	Yes	Yes	No	Yes	73%

**Table 7:** JBI critical appraisal checklist for qualitative studies JBI - Joanna Briggs Institute

Article	Is there congruity between the stated philosophical perspective and the research methodology?	Is there congruity between the research methodology and the research question or objectives?	Is there congruity between the research methodology and the methods used to collect data?	Is there congruity between the research methodology and the representation and analysis of data?	Is there congruity between the research methodology and the interpretation of results?	Is there a statement locating the researcher culturally or theoretically?	Is the influence of the researcher on the research, and vice- versa, addressed?	Are participants, and their voices, adequately represented?	Is the research ethical according to current criteria or, for recent studies, and is there evidence of ethical approval by an appropriate body?	Do the conclusions drawn in the research report flow from the analysis, or interpretation, of the data?	% Appraisal
Pomykala et al., 2008 [28]	Yes	Yes	Yes	Yes	Yes	No	No	Yes	Yes	Yes	80%

The final inclusion and exclusion criteria yielded 30 articles. These 30 articles included nine in general surgery, six in cardiothoracic surgery, five in orthopedic surgery, four in spinal surgery, three in neurosurgery, and three others (otolaryngology, oral/maxillofacial, and gynecologic surgery). A large variety of OMT techniques were performed, as shown in Table 8.

**Table 8:** Characteristics of included studies Abbreviations: BLT=Balanced Ligamentous Tension; CS=Counterstrain; FPR=Facilitated Positional Release; HVLA=High Velocity Low Amplitude; ME=Muscle Energy; MFR=Myofascial Release; ST=Soft Tissue

Surgical Field	Surgery Performed	Author/Year	Study Type	Number of Participants and Demographics	OMT Techniques Utilized	Time Range OMT utilized	OMT Used for a Long Term Complication	Outcome and Findings Post OMT	Limitations
General Surgery	Laparascopic cholecystectomy	Mills et al., 2020 [5]	Case Series	N=9 Females 25-53 years old	ME, HVLA, MFR, FPR, and Articulatory	Unspecified	N/A	— Eight patients (88%) verbally reported symptoms improvement following treatment with OMT at their post-operative appointment — One patient did not experience significant relief after treatment and was referred for a gastroenterology evaluation of persistent symptoms — Three patients without full relief of pain, but with improvement in somatic dysfunction, were referred to an OMT specialist clinic for recurrent dysfunction following intitial treatment — One patient initially reported partial relief of symptoms after OMT but was treated with additional supportive care and stretching exercises without specialist referral — No complications or adverse events were noted following application of OMT	1. Popultion size 2. The use of one osteopath to perform treatments limits external validity 3. Population demographics 4. Potential researcher bias and objective differences in examination or treatment compared with other osteopathic physicians
General Surgery	Various general surgeries with post-op ileus	Baltazar et al., 2013 [11]	Cohort Study	N=55 OMT group (n=17) and non-OMT group (n=38)	Cranial manipulation, Direct MFR, and ME	48 hours post-op	N/A	— No significant difference in times to bowel movement and clear liquid diet between the OMT and non-OMT groups — Mean time to flatus was 3.1 days in the OMT group and 4.7 days in the non-OMT group — Post-operative mean length of stay was significantly different - 6.1 days for the OMT group and 11.5 days for non-OMT group	1. Limited information on specific techniques utilized 2. Unable to establish cause and effect 3. Population size 4. Potential for selection bias due to no standardized use of OMT
General Surgery	Various Open and Laparoscopic Abdominal surgeries with Post-Op ileus	Crow et al., 2009 [14]	Case Series	N=331 OMT group (n=172) and control group (n=139)	Unspecified	Unspecified	N/A	— Shorter length of stay in OMT group — "My patients do better when your team treats them.’’- quote from MD surgeon who usually referred for OMT treatment on post-operative day	1. No standardization of degree of illness 2. Variety of different surgeries performed 3. Large number of OMT administrators 4. Limited information on specific techniques utilized
General Surgery	Post laparoscopic appendectomy with post-op ileus	Domalski et al., 2014 [16]	Case Report	N=1 17 year old female	Subocciptial release, Ribless rib raising, MFR, and Mesenteric lift techniques	Unspecified	N/A	— Patient reported having a bowel movement 20 minutes after treatment — Post-operative ileus was resolved	1. Population size 2. Unspecified days in received post-op OMT 3. Unable to establish cause and effect 4. Lack of comparisons/controls
General Surgery	Major abdominal surgery	Probst et al., 2016 [29]	Randomized Controlled Trial	N=20 Males (n=15) and Females (n=5); 18-81 years old; OMT group (n=10) and Control group (n=10)	Point-of-balance fascial tension for colon, Neuronal inhibition for intestines, and Compression of 4th ventricle technique	1-5 days post-op	N/A	— 13 complications occurred in the OMT group and 18 complications in the control group — For the first 5 post-operative days, in the OMT group, intra-individual pain decreased by a median of 2 on the Numeric Rating Scale (NRS), while pain in the control group remained the same — At the end of the vists, patients in the OMT group had less pain than those in the control group on each post-operative day — Median length of stay was 11.3 days in OMT group and 17.4 days in control group — Time to first stool was 55.2 hours in OMT group compared to 62.8 hours in control group — Patients in the OMT group also had first flatus on post-operative day 1 (POD) and those in the control group on POD 2 — No difference was seen with regard to vomitting in the first 5 post-operative days and post-operative quality of life (PQL) did not differ significantly before surgery, after surgery or on the day of discharge	1. Population size 2. Population demographics 3. A performance and detection bias may be present 4. Inclusion of subgroups such as laparascopic vs open surgery - Inclusion of various surgeries 5. The control group contained no active control of sham intervention 6. The use of one osteopath to perform treatments limits external validity 7. The questionnaire used for evaluation of PQL was extensive and difficult for some patients to answer
General Surgery	Sigmoid colon resection for diverticulitis and inguinal hernia repair	Ridgeway et al., 2010 [31]	Case Report	N=1 55 year old male	ME, Still's, and Articulatory	2 months post-op	N/A	— Decreased pain and tenderness — Increased range of motion — Improved posture	1. Population size 2. Lack of comparison to OMT treatment studies on patients with the same surgery and post-op complaints
General: Bariatric Surgery	Bariatric surgery	Kim et al., 2019 [22]	Randomized Controlled Trial	N=24 Age 18 years and older; Single session of OMT post-op and Morphine PCA (n=12) or morphine PCA only (n=12)	Suboccipital release, Thoracic outlet release, and Rib raising	1 day post-op	N/A	—Less opioid consumption in OMT group 26.9±16.4mg compared to Control 35.1±23.4mg, but it was not statistically significant —No statistical difference in pain scores or median length of stay	1. Population size 2. Lack of detailed population demographics within each group 3. Patient survery could have some bias of pain interpretation
General: Colon and Rectal Surgery	Anorectoplasty due to anorectal malformation at birth	Vismara et al., 2020 [34]	Case Report	N=1 24 month old male	BLT, Balance and hold treatment, CS, and Craniosacral therapy	2 years post-op; One session per month for 4 months and continued sporadic treatments 2 to 3 times per year	After 2 years of constipation	— Increase in defecation frequency — Decreased abdominal pain — More complete bowel evacuation — Patient showed significant improvement after 4 months of treatment — At 4 years old, the patient is experiencing normal function	1. Population size 2. Lack of comparisons utilizing OMT on similar demographic
General Surgery, Orthopedic, and Gynecologic Surgery	Post-op complication relief after appendicitis, diverticulitis, hernia, orthopedic, and gynecologic procedures	Pomykala et al., 2008 [28]	Qualitative	N=94 Additional demographics unspecified	BLT, CS, ST, ME, FPR and Cranial	Unspecified; Single OMT session during hospital stay	N/A	— 42 patients (45%) had less need for pain medications — 71 patients (76%) had less pain — 83 patients (88%) had decreased anxiety/stress — 90 patients (96%) indicated improved recovery — 90 patients (96%) indicated improved comfort	1. Study bias from referring physician 2. Only responses from pts who volunteered survey results 3. Lack of a control group 4. Only given to pts still in the hospital 1 day after treatment
Cardiothoracic Surgery	CABG with median sternotomy	O-Yurvati et al., 2005 [26]	Quasi-Experimental	N=29 Males (n=21) and Females (n=8); OMT group (n=10) and Control group (n=19)	MFR, Lymphatic pump, BLT, Indirect diagphram release, OA decompression, Rib raising, and Sibsons fascial release	During surgery and 1 to 2 hours post-op while patients were unconscious and medically paralyzed	N/A	— Improved thoracic impedance, mixed venous O2 saturation and cardiac index in the group with OMT — Overall OMT provided beneficial hemodynamic changes in cardiac function	1. Population size
Cardiothoracic Surgery	Coronary artery bypass surgery (CABG) with median sternotomy	Roncada, 2020 [32]	Randomized Controlled Trial	N=82 pts Males (n=72) and Females (n=10); OMT group (n=41) and Control group (n=41)	Diaphragm doming, MFR, Suboccipital inhibition, Anterior posterior equilibration, HVLA, MFR, ME, CS, and Functional techniques	4 weeks post-op and continued treatments at 5, 9, and 12 weeks post-op	N/A	— Lower pain intensity at 12 and 52 weeks in OMT group — No significant change in pulmonary function, thoracic stiffness, quality of life or maximum aerobic capacity between groups at 12 weeks or 52 weeks	1. Population size 2. Premature termination due to change in surgical technique
Cardiothoracic Surgery	CABG	Wieting et al., 2013 [35]	Randomized Controlled Trial	N= 53 Males (n=40) and Females (n=13); OMT group (n=17), Placebo group (n=18) and Control group (n=18)	MFR, Rib raising, and ST with suboccipital muscle release	1 day post-op with daily treatments until discharge	N/A	— OMT group had decreased length of stay (p=0.72), earlier return of bowel function (p=0.19), & improved post-operative function (p=0.22)	1. Several different osteopaths/students performing the treatments 2. Limited to surgical patients from a single surgeon
Cardiothoracic Surgery	Excision of a primary pulmonary leiomyosarcoma	Arnold et al., 2010 [10]	Case Report	N=1 56 year old male	ST, MFR, Rib raising, and Pedal lymphatic pump	Immediately post-op twice daily for 3 days	N/A	— Goal was to improve the patient's initial recovery and reduce long-term musculoskeletal dysfunctions associated with a major thoracotomy; however, the outcome was not discussed	1. Population size 2. The outcome of the OMT treatment was not discussed 3. Lack of comparisons
Cardiothoracic Surgery	Thoracoabdominal resection of the esophagus	Bjersa et al., 2013 [13]	Quasi-Experimental	N=8 Males (n=5) and Females (n=3); Mean age 61.9 years old	OA release, Rib raising, Diaphragm doming, Balance of thoracic fascia, and ST	Between 2-7 years post-op and then given once weekly for 10 weeks	Patients with remaining respiratory insufficiency or thoracic pain stiffness with time since surgery varying between 2 to 7 years post-op	— Significantly increased range of motion in the thorax — Positive change in pain — Expiratory vital capacity was only minimally affected by OMT — Participants were generally positive towards OMT	1. Population size 2. Low generalizability 3. Differences in time from procedure
Cardiothoracic Surgery	Posterolateral thoracotomy with lung resection	Fleming et al., 2015 [17]	Case Series	N=38 Males (n=21) and Females (n=17); OMT group (n=23) and Control group (n=15)	MFR, BLT, ME, Rib raising, Articulatory, Cranial, Inhibition, ST, Visceral, Still's, FPR, Neurofascial release, CS, Lymphatic, HVLA, and Integrated neuromusculoskeletal release	Average of 17 days post-op at first treatment and treatment was once weekly for three weeks	N/A	— No statistical difference in length of stay between OMT and non-OMT patients — OMM consultation was more common with pts who had 2 procedures performed during surgery (8 of 12) and with those who were directly admitted to ICU (22 of 23 OMT pts in ICU and 10 of 15 non OMT in ICU)	1. Population size low pt volume, difference in illness severity amongst those in OMT vs non-OMT group, inconsistent timing to OMM consultation/initiation, various (non-standardized) OMT techniques amoung pts
Orthopedic Surgery	Knee arthroplasty, hip arthroplasty, open-reduction internal fixation	Licciardone et al., 2004 [1]	Randomized Controlled Trial	N=60 Males (n=18) and Females (n=42); Above 50 years old; OMT group (n=30) and sham treatment group (n=30)	MFR, CS, ME, ST, HVLA, and Craniosacral manipulation	72 hours after admission to rehab unit; 2-5 sessions weekly, no more than 2 days between sessions	N/A	— Significantly poorer outcomes associated with OMT in the length of stay and rehabilitation efficiency, limited to patients with knee osteoarthritis. Rehab efficiency evaluated with a Function Independence Measure score — Similar outcomes among the OMT and Sham Treatment groups of decreased daily analgesic use during the rehabilitation unit stay, though neither group had significantly greater improvement than the other	1. Condition-specific/surgical-site specific outcome measures were not utilized 2. Utilization of osteopathic students and not fully trained osteopathic physicians to perform OMT
Orthopedic Surgery	Knee arthoplasty, ACL reconstruction	Gugel et al., 2006 [19]	Case Report	N=1 27 year old male	Functional and Indirect methods	1 week post-op; Once monthly for 9 months; Once at a 6 month follow-up	N/A	— Patient able to return to full sports activity in 6 months with OMT — Patient had increased stable mobility	1. Population size 2. Limited information on specific techniques utilized
Orthopedic Surgery	Arthroscopic Meniscectomy	Noblitt et al., 2019 [25]	Case Report	N=1 65 year old female	ME and MFR	6 weeks post-op	N/A	— Somatic dysfunctions were resolved with treatment — Decreased swelling and improved range of motion reported during the 1 week follow-up — Improved ambulation, complete normal range of motion, and no return of swelling reported at 1 month follow-up	1. Population size
Orthopedic Surgery	Right total knee arthroplasty, post-operative narcotic-induced constipation	Shiu et al., 2012 [33]	Case Report	N=1 50 year old male	Mesenteric release	5 days post-op	N/A	— Normal bowel movements same day of surgery — Patient required fewer laxatives throughout inpatient rehab stay	1. Population size
Orthopedic Surgery	Open left rotator cuff repair, post-op singultus secondary to intubation and phrenic nerve block	Petree et al., 2015 [27]	Case Report	N=1 72 year old male	MFR and BLT	6 days post-op	N/A	— Singultus rate slowed tremendously during treatment — 2 days after treatment singultus completely resolved	1. Population size
Spinal Surgery	Lumbar Microdiscectomy	Kim et al., 2017 [21]	Randomized Controlled Trial	N=21 Patients 25–65 years old; Rehabilitation group (n=14) and active control group (n=7)	ST and joint mobilization, MFR, Neuromuscular technique, and ME	2-3 weeks after surgery and continued twice a week for 4 weeks	Patients followed at various points over a 2 year period	— OMT rehabilitation had improved all post-operative outcomes at 2 year follow-up — All post-surgical outcomes in the control group had worsened during the same period — Post-operative physical disability was more improved in the OMT rehabilitation group than in the control group (63% vs. −423%, p<0.05) — Post-operative residual low back pain improved in the OMT rehabilitation group showing a 26% reduction — Low back residual pain had intensified by 5% in the control group — Intensity of post-operative leg pain was reduced by 57% in patients who had received OMT rehabilitation — Leg residual pain had increased by 8% in the control group	1. No true placebo group 2. Unable to patients or physicians from the intervention 3. Bias related to use of self-reported questionnaire 4. Utilization of osteopathic students and not fully trained osteopathic physicians to perform OMT
Spinal Surgery	Lumbar microdiscectomy	Kim et al., 2015 [38]	Randomized Controlled Trial	N=33 Patients 20 - 65 years old; OMT group (n=16) and exercise program group (n=17	ST and joint mobilization, MFR, Neuromuscular technique, ME, Craniosacral release, Rib raising and mobilization	2-3 weeks post-op and continued twice a week for 4 weeks	Patients followed at various points over a 2 year period	— Residual leg pain after the lumbar discectomy decreased in the OMT group with a 53% reduction from baseline compared to the exercise group which had a 17% reduction — Residual low back pain also decreased with 37% reduction in the OMT group and a 10% reduction in the exercise group	1. No true placebo group 2. Unable to blind patients or physicians from the intervention 3. Bias related to use of self-reported questionnaire 4. Utilization of osteopathic students and not fully trained osteopathic physicians to perform OMT
Spinal Surgery	Lumbar open laser microdiscectomy	Kim et al., 2016 [39]	Randomized Controlled Trial	N=21 Patients 25–69 years old; OMT group (n=14) and a control group (n=7)	ST and joint mobilization, MFR, Neuromuscular technique, and ME	2-3 weeks after surgery and continued twice a week for 4 weeks	Patients followed at various points over a 2 year period	— Early post-operative functional disability was more improved by individualised rehabilitation with OMT than active control with self-home exercise (55% vs. −5%, p<0.05) — Reduction in medication use went down 93% in the OMT rehabilitation group and 38% in the control group	1. No true placebo group 2. Unable to patients or physicians from the intervention 3. Bias related to use of self-reported questionnaire 4. Utilization of osteopathic students and not fully trained osteopathic physicians to perform OMT
Spinal Surgery	C2-C4 and C7-T4 bilateral laminectomy and C2-T4 bilateral fusion, with acute muscle spasm	Ivanov et al., 2016 [20]	Case Report	N=1 75 year old female	ME and Indirect MFR	Unspecified; 2 days of therapy	N/A	— Neck pain resolved by 50% — Patient was able to regain much of her range of motion lost	1. Population size
Neurosurgery	Craniotomy for meningioma removal	Berkowitz, 2014 [12]	Case Report	N=1 35 year old female	Vault hold, Frontooccipital hold, Frontal bone lift, Parietal lift, V-spread technique, and Compression of the 4th ventricle	5 weeks post-op	N/A	— Hemianopsia visual field was corrected and remained so for 2 years	1. Population size 2. Lack of comparisons
Neurosurgery	Cervical decompressive laminectomy related to cervical ependymoma	Lewis, 2018 [23]	Case Report	N=1 48 year old female	CS, MFR and FPR	7 months post-op continued to 8.5 months post-op	The patient had increased weakness 7 months post-op	— OMT can assist patients to adapt, compensate, and continue to optimize their functional recovery — OMT can be provided to reduce the patient’s structural and neurologic dysfunction	1. Population size 2. Lack of comparisons
Neurosurgery	Arnold Chiari Type I decompression	Zegarra-Parodi et al., 2010 [36]	Case Report	N=1 29 year old male	Targeted musculoskeletal scar tissue and manual desensitization techniques	5 years post-op	The patient had trigeminal neuralgia 5 years after the decompression surgery	— Clinically significant decrease in overall pain measured by the VAS occured after the second treatment — Increase in cervical function was also reported by the patient	1. Population size 2. Lack of comparisons 3. Limited information on specific techniques utilized
Otolaryngology Surgery	Emergency excision of obstructive granuloma due to blunt laryngeal trauma	Dhanasekar et al., 2006 [15]	Case Report	N=1 55 year old female	ST, Articulatory, and Laryngeal manipulation	6 weeks post-op; Follow up treatment lasted for 15 months	N/A	— OMT assisted in the patient's recovery — 15 months post-injury, her voice had returned to normal — The left vocal cord was only minimally bowed	1. Population size 2. Lack of comparisons 3. Laryngeal manipulation was used in conjunction with voice therapy to assist in recovery
Oral/Maxillofacial Surgery	Excision of right lower third molar	Lipton et al., 2013 [24]	Case Report	N=1 30 year old female	Cranial, Intra-oral ST, and HVLA	10 years post-op; 4 months of treatment which included 9 visits (7 total treatments)	The patient had right jaw pain and dental pain for 10 years after her surgery	— Patient's jaw pain was completely eliminated with the use of OMM — OMM began on her third visit (pain 7/10) and at the 4th visit (second OMM treatment) her pain resolved to a 0/10 — Pain returned at the 5th visit (third OMM treatment) at 3/10, but by the 7th visit (fifth OMM treatment) the patient's pain was a 0/10 and remained resolved for four months and she was subsequently discharged to her PCP	1. Population size 2. Lack of comparisons
Gynecologic Surgery	Total abdominal hysterectomy	Goldstein et al., 2005 [18]	Randomized Controlled Trial	N=39 Females 18 years old or more; Four treatment groups: 1. Pre-operative saline and post-operative sham manipulative treatment (n=9); 2. Pre-operative saline and post-operative OMT (n=10); 3. Pre-operative morphine and post-operative sham manipulative treatment (n=10); 4. Pre-operative morphine and post-operative OMT (n=10)	MFR and ST	Approximately 4 hours after patient returned to their room from the post-anesthesia care unit; At approximately 8am the day after the surgery; At approximately 2pm on the day after the surgery	N/A	— There were no differences in either pain, or nausea and vomitting scores among the four study groups — Patients in the Morphine + OMT group used less morphine than the Morphine + Sham group for the first 24 hours and from 25-48 hours — Morphine blood concentrations in the Morphine + OMT group were also lower after 24 hours compared to the Saline + OMT group — Patients in the Morphine + OMT group weighed more than those in any of the other three study groups — No significant differences detected among the four groups regarding patient demographics such as age and duration of surgery — Administration of post-operative OMT enhanced pre- and post-operative morphine analgesia in the immediate 48-hour period following elective total abdominal hysterectomy — OMT can be a therapeutic adjunct in pain management following elective total abdominal hysterectomy	1. Population Size

General Surgery

In the field of general surgery, nine studies were found that evaluated the impact of OMT in post-operative patients. Three studies were case reports [16,31,34], two were case series [5,14], two were RCTs [22,29], one was a cohort study [11], and one was a qualitative study [28]. While a range of OMT techniques were used across all of the studies, some of the most common were MFR, ME, and cranial manipulation [5,11,14,16,22,29,31,34]. Three of the studies involved post-op ileus [11,14,16], three involved GI resections [11,29,31], and four involved laparoscopic procedures [5,16,22,29]. Many of the studies evaluating the use of OMT after general surgery demonstrated positive outcomes: two demonstrated decreased length of stay in the hospital, four showed significant pain relief, one had a decrease in opioid use, and three exhibited improvements in bowel movement [5,11,14,16,22,29,31,34].

The qualitative study in hospitalized patients utilized balanced ligamentous strain, counterstrain, soft tissue, ME, FPR, and cranial techniques [28]. This study administered a single OMT session during each patient’s hospital stay after surgeries for appendicitis, diverticulitis, hernias, and orthopedic or gynecologic issues and demonstrated patients had less need for pain medication, less pain, decreased anxiety and stress, and improved recovery and comfort [28]. The majority of the surgical procedures in this study were general; therefore, it was most appropriately designated into the general surgery category.

Cardiothoracic Surgery

The search yielded six studies that looked at the use of OMT in patients who were undergoing cardiothoracic surgeries [10,13,17,26,32,35], with three of these being coronary artery bypass (CABG) surgeries [26,32,35], one being a posterolateral thoracotomy [17], one being a thoracoabdominal resection of the esophagus [13], and one being an excision of a primary pulmonary leiomyosarcoma [10]. Some of the most common OMT techniques that were used were MFR, diaphragm doming, rib raising, and ME [10,13,17,26,32,35]. The study by Fleming showed no statistical difference found in length of stay after posterolateral thoracotomy [17]. The study by Arnold did not specifically address the clinical outcome after utilizing OMT but noted the rib raising and pedal lymphatic pump were used to augment lymphatic flow [10]. The post-CABG study by Gert did not show statistically significant changes in pulmonary function, thoracic stiffness, quality of life, or maximum aerobic capacity between the OMT group and the control group. However, the study yielded positive results of decreased pain intensity at 12 and 52-week follow-ups post-CABG [32]. The other post-CABG studies revealed beneficial hemodynamic changes in cardiac function [26], as well as decreased length of stay, earlier return of bowel function, and improved post-operative function [35]. Similar positive results were found for thoracoabdominal resection of the esophagus with decreased pain, decreased thoracic stiffness, and likelihood to recommend OMT to a friend among the OMT groups [13].

Orthopedic Surgery

For the field of orthopedic surgery, four case reports and one randomized controlled trial were identified that looked at the effect of OMT in their post-operative patients. The case reports involved one focusing on upper extremity surgery of an open rotator cuff repair [27], and three focusing on lower extremity surgery of knee arthroplasty ACL repair, arthroscopic meniscectomy, and right total knee arthroplasty [19,25,33]. The one randomized controlled trial involved lower extremity surgeries of knee arthroplasty, hip arthroplasty, and open-reduction internal fixation [1]. The two most common overlapping OMT techniques among these orthopedic studies were ME and MFR [1,19,25,27]. The randomized control trial discussed by Licciardone found significantly poorer outcomes associated with OMT in the length of stay and rehabilitation efficiency, limited to patients with knee osteoarthritis [1]. Rehab efficiency was evaluated with a Function Independence Measure score [1]. It also found similar outcomes among the OMT and Sham treatment groups of decreased daily analgesic use during the rehabilitation unit stay, though neither group had significantly greater improvement than the other [1]. One case report focused on the improvement of post-operative ileus induced by narcotics and found through mesenteric release the patient had returned of normal bowel movement the same day of surgery [33]. The majority of the studies showed an improvement in patient recovery ranging from decreased pain levels reported by the patients using visual analog score, increased range of motion assessed by physical exam via the physician, decreased swelling, improved recovery time, and improvement in post-operative complications of singultus and constipation [19,25,27,33].

Spinal Surgery

Four studies, including three randomized controlled trials [21,38,29] and one case report [20], were specific to spinal surgery, which consisted of lumbar microdiscectomies and a laminectomy. The most commonly utilized OMT techniques among the spinal cases were neuromuscular release, soft tissue, ME, and MFR [21,20,38,39]. The randomized controlled trial studies were part of the same trial over a two-year period of time and looked at the outcomes through different time periods [21,38,39]. The benefits found among the spinal surgery studies were decreased pain levels, reduction in residual back and leg pain, and less usage of pain medication [20,21,38,39].

Neurosurgery

In the field of neurosurgery, three case reports were identified evaluating the usage of OMT following the post-operative period with the following cases: cervical decompression laminectomy for a cervical ependymoma, Arnold Chiari Type I decompression, and a craniotomy for meningioma removal [12,23,36]. Some of the OMT techniques utilized in these studies were cranial techniques, MFR, and counterstrain [12,23,36]. These studies showed OMT benefits of decreased pain, increased functionality, and reduction of neurologic dysfunctions that resulted from the surgeries [12,23,36].

Other Surgical Fields

In other surgical fields not categorized previously, there is otolaryngology, oral/maxillofacial, and gynecological surgery. There was one case report in the field of otolaryngology evaluating the use of soft tissue, articulatory, and laryngeal manipulation OMT following the excision of an obstructing granuloma after blunt trauma [15]. This study showed that the use of OMT aided in the patient's recovery and return to full vocal function [15].

For the field of oral/maxillofacial surgery, there was one case report of a 30-year-old female with continued right jaw and tooth pain ten years after molar excision that was completely eliminated with the use of OMT with techniques of HVLA, intra-oral soft tissue, and cranial manipulation [24].

The one study evaluating the use of OMT in gynecologic surgery was a double-blinded RCT which utilized MFR and soft tissue techniques during the post-operative period [18]. This study showed administration of post-operative OMT enhanced pre- and post-operative morphine analgesia in the immediate 48-hour period following elective total abdominal hysterectomy [18].

Discussion

Summary of Evidence

A total of 30 research articles met our inclusion criteria and were analyzed. The studies were categorized based on their surgical field, and their results and methodology were evaluated. Within each subdivision, a variety of research types were studied ranging from randomized controlled trials to case reports and retrospective charts and case reviews. The most common research type was case reports, which included 14 out of the 30 studies reviewed. The next most reviewed article type was randomized controlled trials, which included nine articles in total. There were nine research articles in general surgery, six in cardiothoracic surgery, five in orthopedic surgery, four in spinal surgery, three in neurosurgery, and three others (otolaryngology, oral/maxillofacial, and gynecologic surgery). The majority of studies demonstrated the benefits utilizing OMT in post-operative patients, with the exception of one randomized controlled trial (RCT) involving OMT after knee and hip surgeries [1]. Despite the lack of benefit found in the aforementioned study, the remaining articles showed a wide range of beneficial outcomes from the use of OMT in post-operative patients.

All of the studies evaluated in the field of general surgery demonstrated positive outcomes. Patients who received OMT after general surgery had decreased length of hospital stay, reduction in pain and somatic dysfunction relief, improved bowel movements, and decreased usage of pain medication post-operatively [5,11,14,16,22,28,29,31,34].

The six cardiothoracic articles also yielded positive results after post-operative implementation of OMT, which included a reduction in pain intensity and need for pain medication, reduced anxiety or stress, beneficial hemodynamic changes, improved recovery and comfort, and decreased length of hospital stay [10,13,17,26,32,35].

The studies reviewed in orthopedic and spinal surgery both showed that the use of OMT in post-operative patients demonstrated reduced pain and swelling, increased range of motion and mobility, and improvements in post-operative complications such as singultus and ileus [19-21,25,27,33,38]. In addition, a spinal surgery study showed early post-operative functional disability showed greater improvement in OMT patients than in-home exercise [39]. Another spinal surgery showed the benefits of OMT two years after the surgery [21].

In the field of neurosurgery, the three studies found showed through the usage of OMT there was correction of hemianopsia, increased cervical function, decreased pain, and reduction of structural and neurologic dysfunctions [12,23,36].

In the category of other surgical fields, which consisted of otolaryngology, oral/maxillofacial, and gynecologic surgery articles, positive effects seen included: return of normal voice after laryngeal trauma, complete resolution of long-term post-operative jaw pain, and decreased opioid use [15,18,24]. As shown above, the benefits of OMT in post-operative patients are well-documented and demonstrate the amenability of OMT in a variety of medical fields from general surgery to neurosurgery.

Limitations

Some of the common limitations among the studies involved were that 14 of the 30 papers were case reports involving only one patient; therefore, a small sample size can make it unclear how those techniques would apply to a larger population [10,12,15,16,19,20,23-25,27,31,33,34,36]. There is also a higher risk of potential bias in case report studies. A small sample population was also an underlying limitation in the rest of the studies found with the largest sample size being 330 patients [14].

Aside from just population size, of the nine randomized controlled trials, some found it difficult to fully blind both the patients and physicians. The OMT techniques are very hands-on and specific, if a physician is blind, they would not be able to fully utilize the proper techniques [21,29,38,39]. For the patients, they would need to not be exposed to OMT in the past to not be able to tell if the “hands-on technique” is truly OMT or sham treatment. While other trials were limited in who provided the OMT treatment, many had only medical students providing which were not fully trained physicians [1,21,35,38,39].

Two retrospective studies and two randomized control trials, which utilized surveys, and one randomized control trial, which had physician referrals, all had the potential for selection bias [5,11,22,28,29]. The randomized control trial by Wieting, had some variation in numbers reported on their published paper on their chart versus their writing, needing the possibility of further evaluation of their data [35].

Another common limitation among many of the studies was the lack of description of the OMT utilized, which can make it difficult to reproduce that study in the future [11,14,19,36]. OMT is also a very personalized technique and plays a role of variability among studies, furthering the possibility of differences in being able to replicate and utilize specific techniques among the different recovery situations.

Finally, there is a limitation with the JBI critical appraisal performed. Ideally, only studies with a low-risk of bias would be included. However, due to the small number of studies identified in the literature search (n=30), it was decided by the research team to include studies with a low risk and a moderate risk of bias. Although there are moderate risk studies included, these studies demonstrate evidence that is beneficial for review given the small amount of literature available. Furthermore, studies with a high risk of bias were not included in the review.

Implications for Research

Future post-operative patients could benefit from additional OMT research in this field of study. This review identified limitations including the lack of comparison, potential selection bias, lack of description for OMT techniques, utilization of medical students, and lack of fully blinding participants. When conducting future research, it would be beneficial to include a detailed description of control groups and the process of patient selection to rule out selection bias and ensure patients have not had prior exposure to OMT before the study. It would also be valuable to describe which OMT techniques are utilized on each patient in detail so that a protocol can be established and utilized by other providers. In addition, uniform reporting of adverse events would be useful as this would allow providers to compare OMT to traditional standards of care utilized in the post-operative setting.

A large randomized controlled trial demonstrating the use of OMT in various surgical fields, while following the above-mentioned variables, could have immense implications for the use of OMT for post-operative patients. Demonstrating the effectiveness of OMT in these patients could also give evidence to the insurance companies that it is a beneficial treatment for post-operative patients, in comparison to traditional standards of care.

## Conclusions

Prior studies have demonstrated numerous examples of OMT effectiveness in various surgical fields, including decreased length of hospital stay, reduction in pain, reduction in pain medication usage, and overall improved recovery and increased comfort. However, there are few studies available in this field, and the studies identified have several limitations, as noted previously. OMT can be beneficial to patients during the post-operative period, and more investigations and literature would further legitimize its implementation into patient care. In addition, patients could benefit from future studies being performed with detailed methodology, descriptions of OMT techniques utilized, and selection biases removed. Providing a guideline for physicians to use with future post-operative patients would be beneficial and would provide patients with the best possible post-operative care.

## Appendices

**Table 9:** Funding sources of references

Funding Source Type	# of Articles (%)
Government-Sponsored	1 (3.33%)
Public-Sponsored	1 (3.33%)
Non-Profit Sponsored	1 (3.33%)
Multiple: Public/Non-Profit/Academic	1 (3.33%)
Non-Sponsored	8 (26.6%)
Not Reported	18 (60.0%)
